# Effect of chromosome substitution on intrinsic exercise capacity in mice

**DOI:** 10.12688/f1000research.3-9.v2

**Published:** 2014-05-28

**Authors:** Sean M. Courtney, Michael P. Massett

**Affiliations:** 1 Department of Health and Kinesiology, Texas A & M University, College Station, TX, 77843-4243, USA; 2 Current address: Department of Surgery; Division of Surgical Oncology, Medical University of South Carolina, Charleston, SC, 29414, USA

## Abstract

Previous research identified a locus on Chromosome 14 as an important regulator of endurance exercise capacity in mice. The aim of this study was to investigate the effect of chromosome substitution on intrinsic exercise capacity and identify quantitative trait loci (QTL) associated with exercise capacity in mice. Mice from a chromosome substitution strain (CSS) derived from A/J and C57Bl/6J (B6), denoted as B6.A14, were used to assess the contribution of Chromosome 14 to intrinsic exercise capacity. All mice performed a graded exercise test to exhaustion to determine exercise capacity expressed as time (min) or work (kg·m). Exercise time and work were significantly greater in B6 mice than B6.A14 and A/J mice, indicating the presence of a QTL on Chromosome 14 for exercise capacity. To localize exercise-related QTL, 155 B6.A14 x B6 F _2_ mice were generated for linkage analysis. Suggestive QTL for exercise time (57 cM, 1.75 LOD) and work (57 cM, 2.08 LOD) were identified in the entire B6.A14 x B6 F _2_ cohort. To identify putative sex-specific QTL, male and female F _2_ cohorts were analyzed separately.  In males, a significant QTL for exercise time (55 cM, 2.28 LOD) and a suggestive QTL for work (55 cM, 2.19 LOD) were identified.  In the female cohort, no QTL was identified for time, but a suggestive QTL for work was located at 16 cM (1.8 LOD). These data suggest that one or more QTL on Chromosome 14 regulate exercise capacity. The putative sex-specific QTL further suggest that the genetic architecture underlying exercise capacity is different in males and females.  Overall, the results of this study support the use of CSS as a model for the genetic analysis of exercise capacity. Future studies should incorporate the full panel of CSS using male and female mice to dissect the genetic basis for differences in exercise capacity.

## Introduction

Cardiorespiratory fitness measured during a graded exercise test is inversely related to the relative risk of cardiovascular disease ^1, 2^. Results from human cross-sectional, twin, and prospective studies indicate that genetic factors account for 25–65% of the variation in exercise capacity ^3, 4^. Because having higher levels of exercise capacity has been shown to be beneficial for reducing the onset of cardiovascular disease, the physiological factors determining exercise capacity have been widely studied ^5^. However, the genetic contribution to exercise capacity is not completely understood. Presently, several candidate genes contributing to improved exercise capacity have been proposed based on genome wide studies ^6, 7^, but these genes account for only a small portion of the variability in exercise capacity or training responses ^8^.

Several studies have investigated the genetic factors contributing to exercise capacity using inbred rodent models ^9– 13^. One common approach has been to screen multiple rodent strains for exercise capacity, followed by quantitative trait loci (QTL) analyses to identify loci linked to exercise capacity. This approach has been used to identify QTL for exercise capacity in rats ^10^ and mice ^11, 13, 14^. Research from our laboratory previously identified significant and suggestive QTL on several chromosomes that may house candidate genes that influence variation in exercise capacity ^13, 14^. These identified regions overlap with other mouse and human QTL, suggesting that these regions and/or genes are conserved among species ^13^. Mouse Chromosome 14 (Chr 14), for example, contained a significant QTL for intrinsic (pre-training) exercise capacity, a significant QTL for exercise capacity after training, and a suggestive QTL for the change in exercise capacity in response to exercise training ^13^. Several linkage markers for maximal oxygen consumption in the sedentary state in humans map to these exercise-related QTL on mouse Chr 14 ^13, 15^. Therefore, the present study focused on characterizing the role of Chr 14 in regulating intrinsic exercise capacity.

In the current study we employed a relatively new mouse model, chromosome substitution strains (CSS) to assess the contribution of individual chromosomes to endurance exercise capacity ^16, 17^. CSS mice are made by substituting a single chromosome from a donor inbred strain on the genetic background of a host inbred strain (recipient). Therefore phenotypic differences between the recipient or background strain mice and CSS mice support the presence of a QTL on the substituted chromosome for the phenotype being measured. Results from a previous study identified the A/J strain as having low exercise capacity in comparison to the C57BL/6J (B6) strain ^14^. Therefore we chose to use CSS mice based on A/J and B6 inbred strains.

Utilizing this CSS model, the main purposes of the present study were to investigate the effect of chromosome substitution on intrinsic exercise capacity and to identify QTL regulating intrinsic exercise capacity in mice. We hypothesized that chromosome substitution would significantly affect exercise capacity and therefore confirm the importance of Chr 14 in the genetic regulation of intrinsic exercise capacity in mice. Furthermore, we utilized linkage analysis to map QTL on Chr 14 in progeny from a cross between the CSS and host B6 strain.

## Methods

### Animals

All procedures adhered to the established National Institutes of Health guidelines for the care and use of laboratory animals and were approved by the Institutional Animal Care and Use Committee at Texas A&M University. Seven week-old inbred mice (A/J, C57BL/6J (B6), and Chr 14 substitution mice (C57BL/6J-Chr 14 ^A/J^/NaJ, abbreviated B6.A14)) (n = 12/strain, 6 male and 6 female mice) were purchased from Jackson Laboratory (Bar Harbor, ME.). Upon arrival at Texas A&M, all mice were given one week to acclimatize to their new environment before assessing exercise capacity. A separate group of male B6 mice were crossed with a separate group of female chromosome substitution B6.A14 mice to generate (B6.A14 × B6) F _1_ mice. The F _1_ mice were then intercrossed to produce 155 F _2_ generation mice (67 male and 88 female mice). All mice were housed in standard hanging polycarbonate cages (43 cm long × 21.5 cm wide × 15 cm high) with hardwood chip bedding and allowed food (Standardized Laboratory Rodent Diet) and water *ad libitum*. Mice were housed 1–5 mice per cage depending on sex and lineage and maintained on a 12 hr light:dark schedule at an ambient temperature of 22–24°C.

### Exercise performance test

At 8 weeks of age, all mice were familiarized for two days at 9.0 m/min and 10.0 m/min at 10° for 10 minutes to run on a motorized rodent treadmill (Columbus Instruments, Columbus, OH), with an electric grid (160 V, 0–2 mA) at the rear of the treadmill as described previously ^13, 14^. Each mouse then completed two graded exercise tests separated by 48 hrs. Mean values for each mouse were used for statistical analyses. For each performance test, the treadmill was started at 9.0 m/min at 0° grade for 9 minutes as a warm-up. The grade was then increased 5° every 9 minutes up to a final grade of 15° and speed was increased 2.5 m/min from a starting speed of 10 m/min every three minutes until exhaustion. Exercise continued until each mouse refused to run, defined as an inability to maintain running speed in spite of repeated contact with the electric grid ^13, 14^. At exhaustion, each mouse was immediately removed from the treadmill and returned to its home cage. Exercise capacity was estimated for each animal using time (minutes) and work (kg·m). Work performed (kg·m) or vertical work was calculated as a product of body weight (kg) and vertical distance (meters), where vertical distance = (distance run)(sinθ), where θ is equal to the angle of the treadmill from 0° to 15° ^13, 14^.

### Genotyping

At least 24 hours after the last graded exercise test, all mice were anesthetized by intraperitoneal injection of a ketamine (80 mg/kg) - xylazine (5 mg/kg) cocktail. Mice were subsequently euthanized by exsanguination due to removal of the heart and aorta. Heart, gastrocnemius, plantaris, soleus muscle and liver tissue were excised from mice, washed in ice-cold (4°C) saline, blotted dry to remove excess liquid, and snap frozen in liquid nitrogen. DNA was isolated from 25 mg of liver tissue with a DNeasy Blood and Tissue kit (Qiagen Science, Germantown, Maryland) according to the manufacturer’s instructions and quantified using NanoDrop spectrophotometry. Genotyping was performed using competitive allele-specific polymerase chain reaction (PCR) single nucleotide polymorphism (SNP) genotyping (KBiosciences, Hoddesdon, UK) ^13^. All 155 F _2_ mice were genotyped using 12 SNPs spaced at approximately 5 cM intervals ^18^.

### QTL identification

QTL analyses were performed using R/qtl ^19^. One-dimensional scans were performed on the entire F _2_ cohort with no additional covariates and with sex included as an additive and interactive covariate ^20^. Permutation tests (1,000 repetitions) were used to identify threshold values for logarithm of odds (LOD) scores for each condition (i.e., with or without covariates) and exercise phenotype ^21^. LOD scores were defined as significant if they surpassed the P < 0.05 threshold and suggestive if they surpassed the P < 0.63 threshold. If suggestive or significant QTL were identified using sex as interactive covariate, then one-dimensional scans were performed on male and female mice separately to identify potential sex-specific QTL. A two-dimensional scan also was performed on the entire F _2_ cohort to identify additive or interacting QTL on Chr 14. QTL confidence intervals were determined using the 1.5 LOD support interval ^19^.

### Statistics

All data are represented as mean ± SE. Statistical significance for phenotype comparisons was denoted by P < 0.05. Two-way analysis of variance was used to determine the effect of sex and strain on exercise capacity, which is defined as time (minutes), or work (kg·m) (JMP 9.0, SAS, Cary, NC). If significant main effects were found for strain, Dunnett’s post hoc test was used to determine significant strain differences compared with B6. If significant main effects were found for sex, t-tests were used to identify sex differences within each strain. Comparisons among parental strains and F _2 _offspring and across genotypes for allelic effects were made using one way analysis of variance (strain or genotype) followed by Tukey’s post-hoc analysis. T-tests were used to identify sex differences in F _2_ offspring. Linear regression was used to determine the contribution of body mass to exercise performance.

## Results

**Figure 1.**
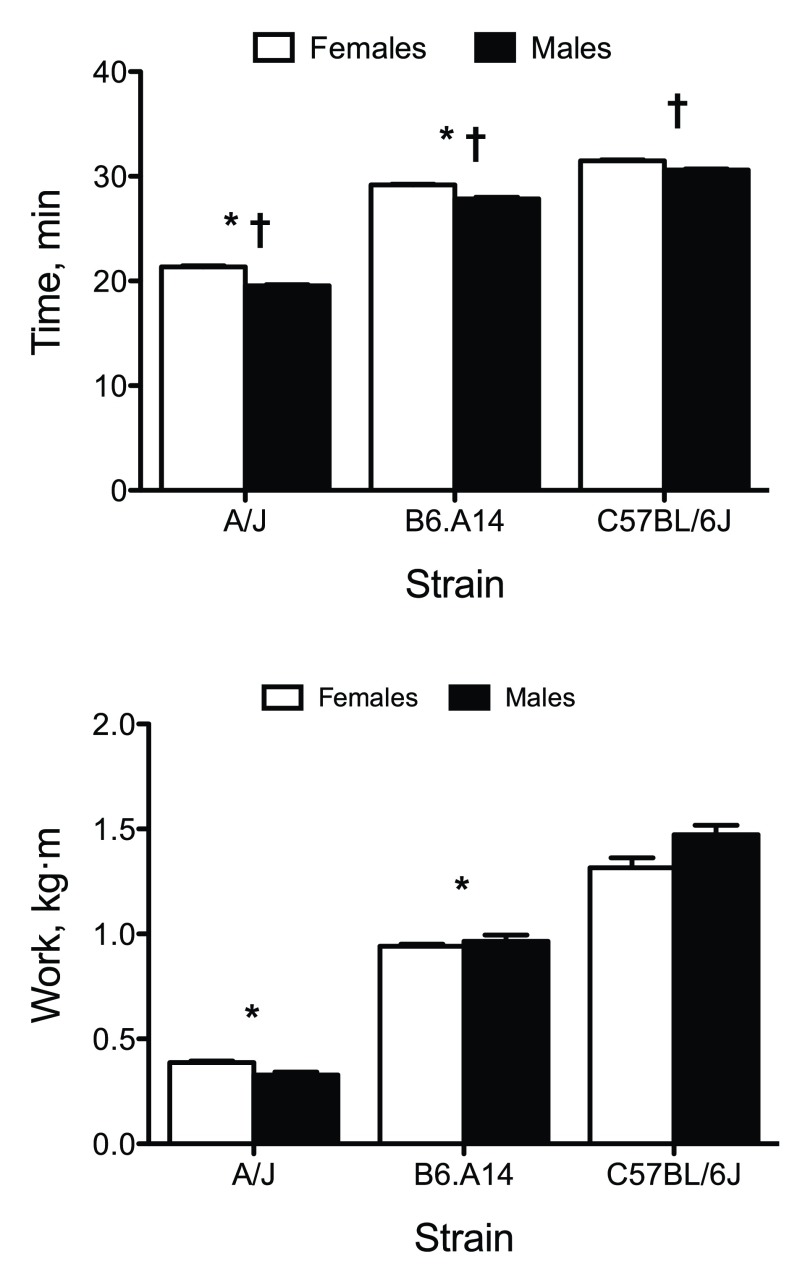
Strain- and sex-dependent differences in exercise capacity in male and female mice from A/J, C57BL/6J (B6), and B6.A14 chromosome substitution strains. Exercise capacity was assessed using a graded exercise test and expressed as ( **A**) time in minutes or ( **B**) work in kg·m. Values are expressed as mean ± SE. n = 6 mice per group. *P < 0.05 compared to B6; ^†^P < 0.05 compared to females.

*Inbred strains.* Exercise capacity, defined as mean run time during two graded exercise treadmill tests, for inbred and CSS mice is shown in Figure 1. Exercise times in A/J and B6.A14 mice were significantly less (P < 0.0001) than that in B6 mice. A significant effect of sex also was identified in all strains (A/J, B6.A14, and B6). For each strain, female mice ran significantly longer than male mice from the same strain ( Figure 1A). When exercise capacity was expressed as work, A/J and B6.A14 strains were significantly different from B6 (P < 0.0001), with mice from both strains performing less work than B6 mice ( Figure 1B). In contrast to exercise time, there was no significant main effect for sex (P = 0.1) on exercise capacity defined as work. Significant differences among the strains were primarily limited to differences in exercise phenotypes. Body mass was significantly less in B6.A14 mice compared with B6 (P < 0.0008) ( Table 1). There were no significant differences in absolute tissue mass among A/J, B6, and B6.A14 strains ( Table 1). Within each strain, body mass was significantly lower in females compared to males. Accordingly, tissue masses were lower in female mice compared to male mice from the same strain ( Table 1). For each strain there was a significant negative correlation between body mass and exercise time (B6, r = -0.71, P = 0.0096; A/J, r = -0.77, P = 0.0035; B6.A14, r = -0.86, P = 0.0003).

**Table 1.** Physical characteristics of female and male inbred and B6.A14 CSS mice.

	A/J		B6.A14		C57BL/6J	
	female	male	female	male	female	male
Body mass, g	19.3 ± 0.2 ^†^	22.7 ± 0.8	17.1 ± 0.0 ^†^	20.7 ± 0.4*	18.5 ± 0.6 ^†^	22.8 ± 0.7
Heart mass, mg	94.0 ± 3.5 ^†^	105.8 ± 3.6	93.7 ± 2.0 ^†^	116.0 ± 3.5	104.3 ± 2.8 ^†^	122.0 ± 5.9
HM:BM, mg/g	4.94 ± 0.17	4.63 ± 0.10	5.44 ± 0.12	5.55 ± 0.08*	5.68 ± 0.21 ^†^	5.03 ± 0.13
Gastrocnemius mass, mg	120.0 ± 4.2	124.0 ± 3.1	99.8 ± 4.6 ^†^	130.0 ± 2.8	113.3 ± 3.4 ^†^	151.8 ± 6.0
GM:BM, mg/g	6.30 ± 0.21	5.44 ± 0.20	5.79 ± 0.27	6.23 ± 0.18	6.18 ± 0.29	6.27 ± 0.15
Soleus mass, mg	7.7 ± 0.3	8.5 ± 0.5	8.0 ± 0.4	8.3 ± 0.3	7.5 ± 0.3 ^†^	9.5 ± 0.2
SM:BM, mg/g	0.40 ± 0.02	0.37 ± 0.02	0.46 ± 0.02 ^†^	0.40 ± 0.01	0.41 ± 0.03	0.40 ± 0.01
Plantaris mass, mg	21.0 ± 1.1	22.7 ± 1.1	17.8 ± 0.3 ^†^	21.0 ± 1.0	17.8 ± 0.4 ^†^	20.3 ± 0.4
PM:BM, mg/g	1.10 ± 0.05	0.99 ± 0.05*	1.03 ± 0.02	1.01 ± 0.05*	0.97 ± 0.04 ^†^	0.84 ± 0.02

Values are mean ± SE. n = 6 per group, except n = 5 for B6 males. HM:BM, heart mass-to-body mass ratio; GM:BM, gastrocnemius mass-to-body mass ratio; SM:BM, soleus mass-to-body mass ratio; PM:BM, plantaris mass-to-body mass ratio.

*P < 0.05 significant main effect for strain compared with C57BL/6J.

^†^P < 0.05 significantly different from male mice of same strain.

*(B6.A14 × B6) F _2_ mice*.

**Figure 2.**
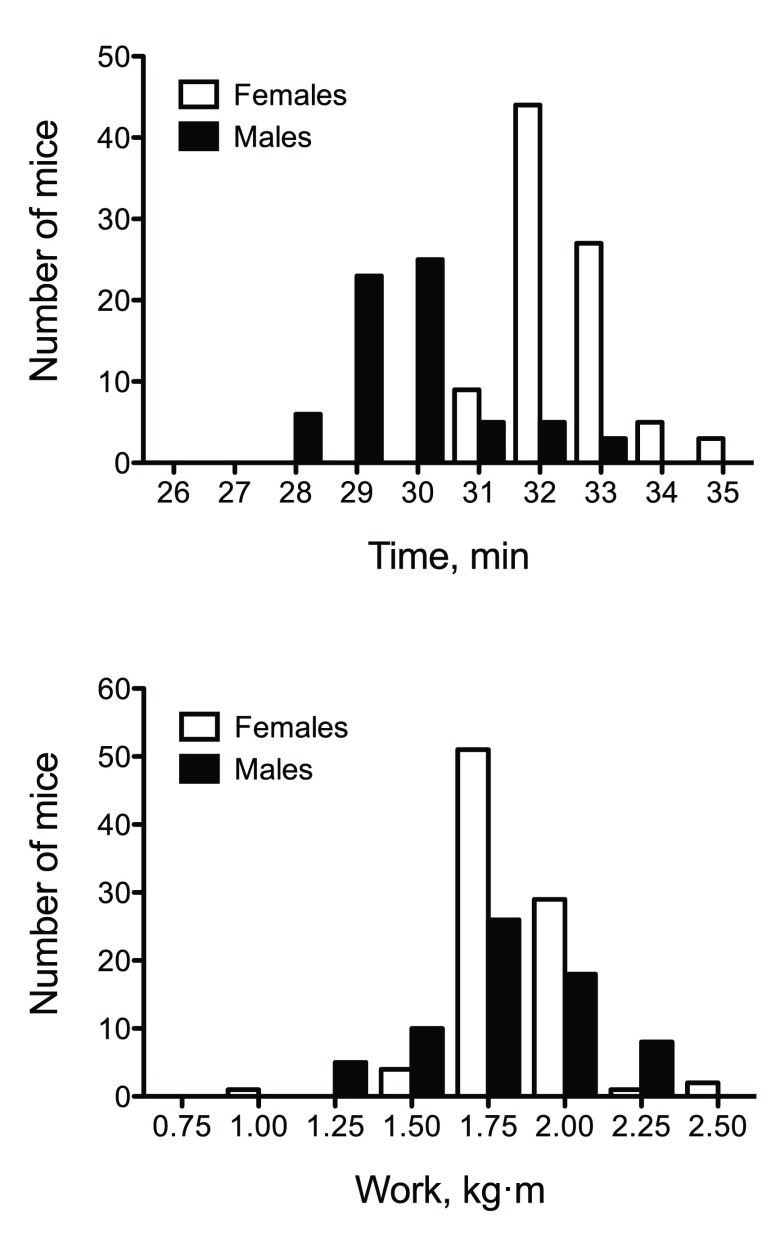
Frequency distribution for ( **A**) time and ( **B**) work in male and female (B6.A14 × B6) F _2_ mice. All F _2_ mice (n = 155) performed a graded exercise test to exhaustion to assess exercise capacity. Mice assorted into 1min/0.25 kg·m buckets. n = 67 for males and n = 88 for females.

The sex-specific distributions for exercise time and work in F _2_ mice are shown in Figure 2. Both time and work varied significantly between male and female F _2_ mice ( Table 2). On average, female mice ran approximately 2.5 min longer than male mice. These differences in run time between male and female mice were offset by a significantly higher body mass in male mice (P < 0.0001) resulting in comparable levels of work in male and female mice. Body mass was approximately 5 g higher in male mice compared with females (P < 0.0001), which likely accounts for the similar levels of work performed. Similar to body mass, tissue masses were significantly smaller in female F _2_ mice compared to their male counterparts.

**Table 2.** Exercise capacity and physical characteristics of female and male (B6.A14 × B6) F _2_ mice.

	Female (n = 88)	Male (n = 67)
Time, min	31.9 ± 0.1 *	29.3 ± 0.1
Work, kg·m	1.70 ± 0.02	1.68 ± 0.03
Body mass, g	19.9 ± 0.2 *	25.0 ± 0.2
Heart mass, mg	110.8 ± 1.3 *	129.3 ± 1.6
HM:BM, mg/g	5.43 ± 0.04 *	5.13 ± 0.05
Gastocnemius mass, mg	127.7 ± 1.4 *	155.8 ± 1.8
GM:BM, mg/g	6.28 ± 0.07	6.19 ± 0.06
Soleus mass, mg	9.1 ± 0.1 *	9.5 ± 0.1
SM:BM, mg/g	0.45 ± 0.01 *	0.38 ± 0.01
Plantaris mass, mg	19.2 ± 0.3 *	22.1 ± 0.3
PM:BM, mg/g	0.94 ± 0.01 *	0.87 ± 0.01

Values are mean ± SE. HM:BM, heart mass-to-body mass ratio; GM:BM, gastrocnemius mass-to-body mass ratio; SM:BM, soleus mass-to-body mass ratio; PM:BM, plantaris mass-to-body mass ratio. *, P < 0.05 significantly different from male mice.

Relative to the progenitor strains, eight week old F _2_ mice ran an average of 30.8 ± 0.1 min, which was significantly longer (P < 0.0001) than B6.A14 (28.5 ± 0.2 min) and not different from B6 (31.0 ± 0.1 min) mice. F _2_ mice also performed significantly more work (1.69 ± 0.02 kg·m, P < 0.0001) than B6 (1.39 ± 0.04 kg·m) and B6.A14 (0.95 ± 0.02 kg·m) strains. Body mass also was compared across strains and generations. F _2_ mice had average body mass of 22.1 ± 0.2 g, which was significantly greater than (P < 0.0001) the progenitor B6 (20.7 ± 0.8 g), and B6.A14 (18.9 ± 0.6 g) strains, respectively. Similar to the inbred strains, there was a significant negative correlation between exercise time and body mass (r = -0.74, P = 0.02). However, this relation was not maintained when the F _2_ population was divided by sex. In male F _2_ mice the correlation between exercise time and body mass was -0.47 (P = 0.001), but there was no significant relation between these variables in female F _2_ mice (r = 0.14, P = 0.18).

**Figure 3.**
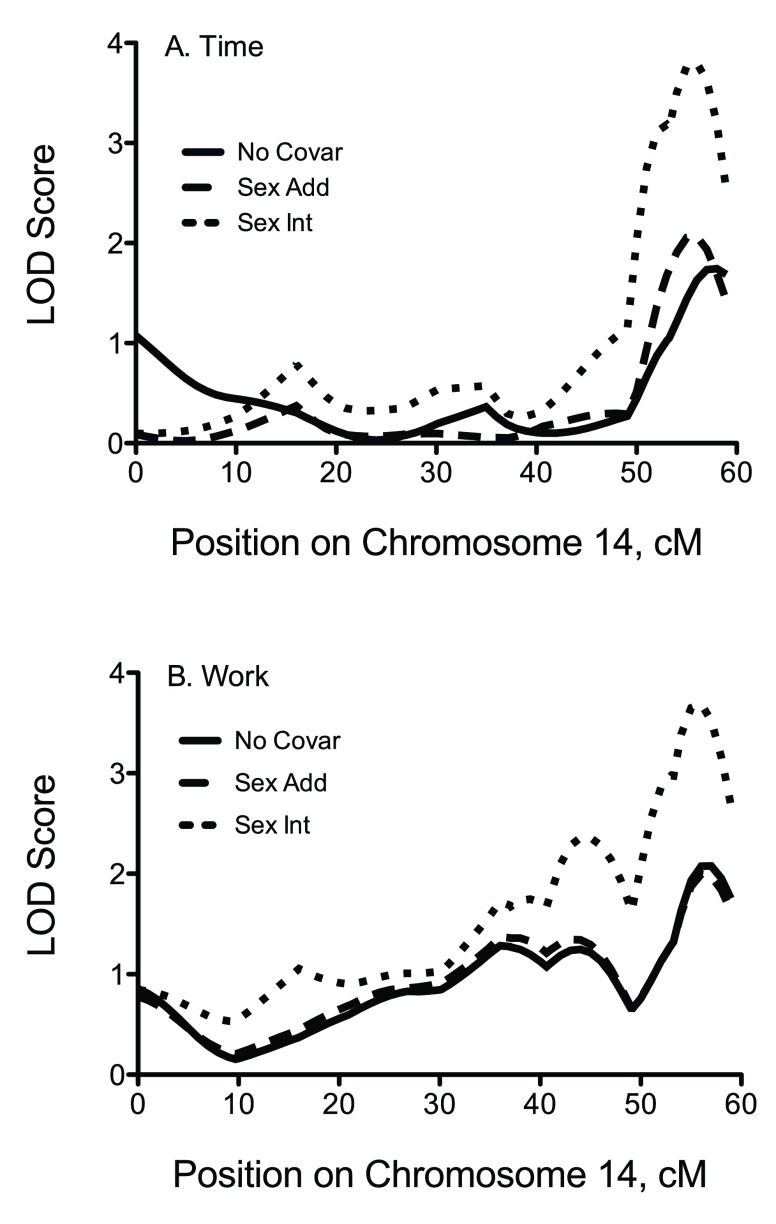
QTL analyses on mouse Chromosome 14 for intrinsic capacity expressed as time (min, **A**) and work (kg·m, **B**) in 155 (B6.A14 × B6) F _2_ mice. Three analyses were performed for each phenotype: 1) with no covariates, 2) with sex as an additive covariate, and 3) with sex as an interactive covariate. For time, significant (P = 0.05) logarithm of odds (LOD) thresholds are 2.22 with no covariates, 2.12 with sex as an additive covariate, and 3.78 with sex as an interactive covariate. For work, significant (P = 0.05) LOD thresholds are 2.22 with no covariates, 2.27 with sex as an additive covariate, and 3.95 with sex as an interactive covariate. LOD thresholds were determined using 1000 permutations. Chromosome-wide scans and permutation analyses were performed using R/qtl.

**Figure 4.**
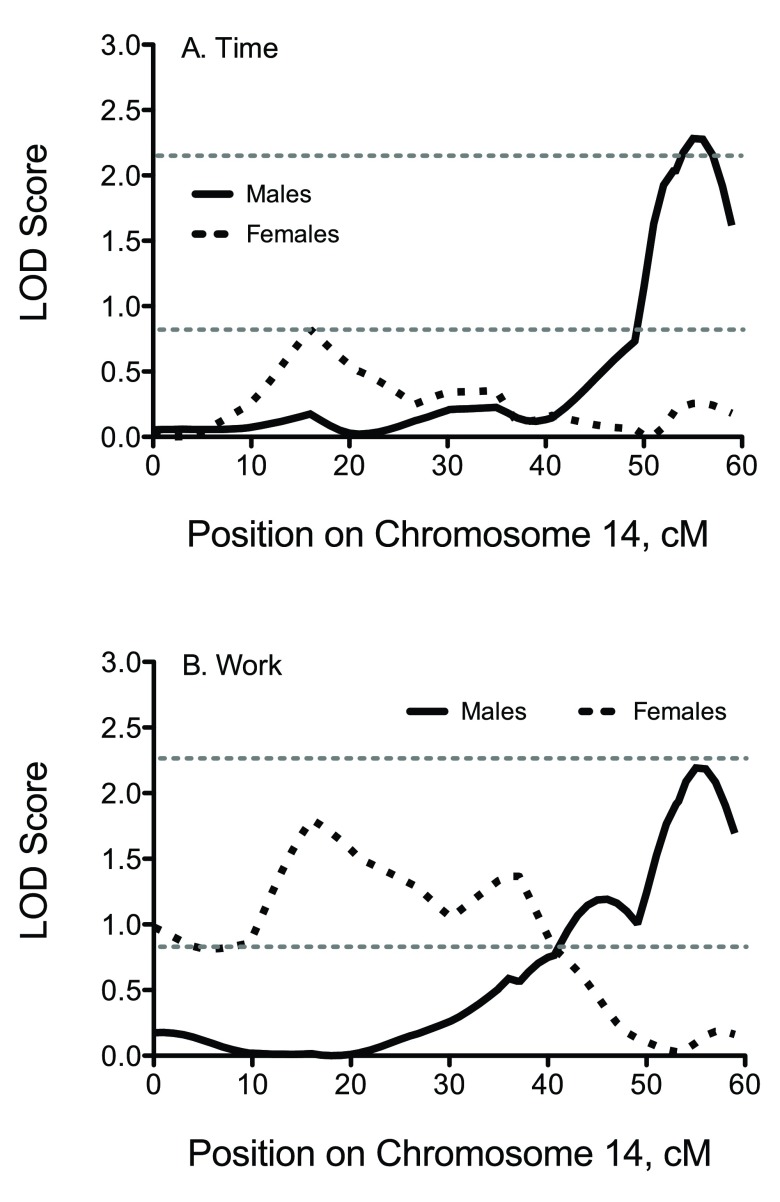
QTL analyses for the effect of sex on intrinsic exercise capacity in male and female (B6.A14 × B6) F _2_ mice expressed as time ( **A**) and work ( **B**). Single chromosome-wide scans for time (in min) and work (in kg·m) were performed separately on male and female F _2_ mice. Dashed lines in the upper graph represent the suggestive (0.82, P = 0.63) and significant (2.21, P = 0.05) logarithm of odds (LOD) thresholds for time in males. Dashed lines in the lower graph represent the suggestive (0.82, P = 0.63) and significant (2.25, P = 0.05) LOD thresholds for work in males. In females, suggestive and significant LOD thresholds for time were 0.84 (P = 0.63) and 2.15 (P = 0.05), respectively; and for work 0.81 (P = 0.63) and 2.08 (P = 0.05), respectively. LOD thresholds were determined using 1000 permutations. Chromosome-wide scans and permutation analyses were performed using R/qtl.

*QTL analysis*. Significant differences in exercise capacity between B6.A14 and B6 strains indicate the presence of a QTL on Chr 14. To fine-map the QTL, F _2_ mice were generated from B6.A14 and B6 strains. Single chromosome-wide scans for time and work are shown in Figure 3. Suggestive QTL for time (LOD = 1.75, P = 0.131) and work (LOD = 2.08, P = 0.063) with no covariates were identified on Chr 14. When sex was included as an interacting covariate, a significant QTL was identified at 56 cM for time (LOD = 3.8, P = 0.048) ( Figure 3A). A suggestive QTL for work (LOD = 3.69, P = 0.07) was identified at the same location ( Figure 3B). Because significant and suggestive QTL were identified using sex as an interacting covariate, chromosome-wide scans were performed on male and female mice separately. In male mice, a significant QTL for time (LOD = 2.28, 1.5 LOD = 49.0 – 58.9 cM, P = 0.049) and a suggestive QTL for work (LOD = 2.19, 1.5 LOD = 38.0 – 58.9 cM, P = 0.056) were identified at 55 cM. In female mice, no QTL were identified for time ( Figure 4A). However, a suggestive QTL for work was identified at 16 cM (LOD = 1.8, P = 0.106) ( Figure 4B). The two-QTL analyses for time showed limited evidence for additive QTL at 0 cM and 58 cM (LOD = 2.74, P = 0.19) on Chr 14. No significant additive or interacting QTL were identified for work. QTL scans also were performed for all physical characteristics and no significant QTL were identified.

The allelic effects for suggestive and significant QTL are shown in Table 3. In the entire F _2_ cohort, heterozygous mice had the highest average exercise time and work. For both phenotypes there was no significant difference between homozygous A and B groups. A similar pattern was observed for time and work in the male F _2_ cohort. In this group, mice with parental genotypes had significantly lower exercise time than mice carrying the heterozygous genotype ( Table 3). In the female F _2_ cohort, work was significantly higher in homozygous A mice compared with homozygous B mice. Heterozygous female F _2_ mice had an intermediate phenotype.

**Table 3.** Allelic effects for significant and suggestive QTL for exercise time and work in sex-specific and entire F _2_ cohorts.

Cohort	Trait	Position, cM	Marker	Genotype			p-value		
				A	H	B	A vs. B	A vs. H	B vs. H
All F _2_	Time, min	58	rs3685710	30.4 ± 0.3	31.1 ± 0.2	30.4 ± 0.3	0.986	0.078	0.067
	Work, kg·m	57	rs3685710	1.68 ± 0.03	1.74 ± 0.02	1.63 ± 0.03	0.532	0.244	0.019*
Males	Time, min	55	rs3715673	28.9 ± 0.2	29.7 ± 0.3	28.8 ± 0.2	0.945	0.048*	0.042*
	Work, kg·m	55	rs3715673	1.62 ± 0.05	1.76 ± 0.04	1.59 ± 0.08	0.933	0.107	0.078
Females	Work, kg·m	16	rs3696080	1.78 ± 0.05	1.72 ± 0.02	1.65 ± 0.03	0.035*	0.530	0.107

Position, location of peak marker in cM; Marker, SNP marker closest the LOD peak; Genotype, genotype at peak marker; A, homozygous for A/J allele; B, homozygous for B6 allele; H, heterozygous; p-value, p-value from means comparison using Tukey post-hoc analysis with specific allelic comparisons indicated (e.g., A vs. B), significant p-values are indicated by*.

Effect of chromosome 14 substitution on intrinsic exercise capacity in mice: R/qtl linkage analysis and phenotype data B6A_CSS_F2.csv: File contains data formatted for use in R/qtl for linkage analysis. The first row contains the phenotype and SNP marker names. In the second row, chromosome numbers are indicated in the genotype columns. These cells are blank in the phenotype columns. The third row contains the position in centiMorgans of the SNP markers. These cells are blank in the phenotype columns. ID, mouse ID number; Strain, mouse strain; pgm, paternal grandparent; body mass, body mass in g at testing; time, exercise time in min; distance, distance run in meters; work, work performed during exercise test in kg•m; tbw, terminal body weight (g); heart, heart weight (mg); hwbw, heart weight to body weight ratio (mg/g); gastroc, gastrocnemius muscle mass (mg); rgbw, gastrocnemius weight to body weight ratio (mg/g); soleus, soleus muscle weight (mg); rsbw, soleus muscle weight to body weight ratio (mg/g); plantaris, plantaris muscle weight; rpbw, plantaris muscle weight to body weight ratio (mg/g); sex, female = 0, male = 1; SNPs used for genotyping, rs47601255, rs3719262, rs3696080, rs3697794, rs3660830, rs3023675, s3706792, rs3676913, rs3684516, rs30501790, rs3715673, rs3685710. Genotypes are indicated by AA for homozygous A/J, BB for homozygous B6, and AB for heterozygous. Body mass, time, distance, and work phenotypes are the mean values of two trials. Data were used to generate Figures 3 and 4 and Table 3 in the main article.
F2_data.csv: Phenotype data for CSS F2 mice used to generate Figure 2 and Tables 2 and 3 in the main article. ID, mouse ID number; SEX, female = 0, male = 1; BW1, body mass in g at test 1; BW2, body mass in g at test 2; BW_mean, average body mass of test 1 and 2; Time1, exercise time in min during test 1; Time2, exercise time in min during test 2; Time_mean, average time of tests 1 and 2; Distance1, distance run in m during test 1; Distance2, distance run in m during test 2; Distance_mean, average distance run during tests 1 and 2; Work1, work performed in kg•m during test 1; Work2, work performed in kg•m during test 2; Work_mean, average work performed during tests 1 and 2; HEART (mg), heart weight (mg); H/BM, heart weight to body weight ratio (mg/g); RG (mg), gastrocnemius muscle mass (mg); RG/BM, gastrocnemius weight to body weight ratio (mg/g); RS (mg), soleus muscle weight (mg); RS/BM, soleus muscle weight to body weight ratio (mg/g); RP (mg), plantaris muscle weight; RP/BM, plantaris muscle weight to body weight ratio (mg/g).
CSS_data.csv: Phenotype data for inbred male and female A/J, C57BL/6J, and B6.A14 mice. Data were used to generate Figure 1 and Table 1 in the main article. ID, mouse ID number; Strain, mouse strain; SEX, female = 0, male = 1; BW1, body mass in g at test 1; BW2, body mass in g at test 2; BW_mean, average body mass of test 1 and 2; Time1, exercise time in min during test 1; Time2, exercise time in min during test 2; Time_mean, average time of tests 1 and 2; Distance1, distance run in m during test 1; Distance2, distance run in m during test 2; Distance_mean, average distance run during tests 1 and 2; Work1, work performed in kg•m during test 1; Work2, work performed in kg•m during test 2; Work_mean, average work performed during tests 1 and 2; HEART (mg), heart weight (mg); H/BM, heart weight to body weight ratio (mg/g); RG (mg), gastrocnemius muscle mass (mg); RG/BM, gastrocnemius weight to body weight ratio (mg/g); RS (mg), soleus muscle weight (mg); RS/BM, soleus muscle weight to body weight ratio (mg/g); RP (mg), plantaris muscle weight; RP/BM, plantaris muscle weight to body weight ratio (mg/g).

## Discussion

The purpose of the current study was to determine the role of mouse Chr 14 in the genetic regulation of exercise capacity and to fine map this chromosome to identify QTL for exercise capacity. Significant differences in exercise time and work were observed between inbred B6 mice and mice carrying Chr 14 from the A/J strain on the B6 background (B6.A14). These differences suggest the presence of one or more QTL on Chr 14 underlying variation in exercise capacity. Utilizing a (B6.A14 × B6) F _2_ population, suggestive QTL for exercise time and work were localized to a position of ~58 cM on Chr 14. Further analysis revealed putative sex-specific QTL for exercise time and work. QTL identified in the male cohort was similar to that in the entire F _2_ cohort, but the suggestive QTL for work identified in the female F _2_ mapped to an alternative position. Collectively, these data suggest that one or more genes on Chr 14 contribute to variation in exercise capacity and that the genetic architecture for exercise-related traits might be different in males and females. Given the complexity of the trait, genome-wide mapping strategies should be employed to identify additional QTL underlying the variation in exercise capacity.

B6 and A/J strains show significant phenotypic differences across many traits ^16, 22– 25^. We, and others ^14, 26, 27^ have demonstrated that exercise capacity assessed by treadmill running is one of these traits. Although testing protocols varied, A/J mice repeatedly show low exercise capacity, having running times approximately 60% or less than that of B6 mice. In the current study, A/J mice ran 10 minutes less than B6 mice and performed only 25% of the work of B6 mice during a graded exercise test ( Figure 1). These observations replicated our previous finding that A/J mice had the lowest exercise capacity among 34 strains tested ^14^. Although B6 were in the lowest third of that survey, their run time was about 60% higher than A/J mice. This disparity in exercise capacity between inbred strains suggests that genetic variation contributes to these phenotypic differences.

Chromosome substitution strains were developed to facilitate genetic analysis of complex traits by partitioning the genome into individual chromosomes ^16^. Phenotypic differences between a CSS and the background strain suggest the presence of at least one QTL on the substituted chromosome. To begin to identify the genetic factors contributing to variation in exercise capacity, mice from a chromosome substitution strain based on A/J and B6 strains were used. In the current study we focused on Chr 14 because we had previously identified several exercise-related QTL on this chromosome ^13^. B6.A14 mice had significantly lower exercise capacity expressed as time or work compared with B6 mice ( Figure 1). Exercise time was 2.5 minutes less in B6.A14 mice, which corresponds to 24% of the difference between parental A/J and B6 strains (10.6 min). The difference in work was 0.44 kg·m, which is approximately 43% of the difference between parental strains (1.03 kg·m). Although the substituted chromosome shifted the phenotype toward the donor strain, the effect of chromosome substitution on exercise capacity was less than expected. Based on previous CSS surveys, chromosome substitution can produce phenotypic effects of 75% or more of the difference between parental strains ^16, 23, 24^. Nevertheless, the significant difference between B6 and B6.A14 for exercise time and work suggest the presence of one or more QTL on Chr 14 for exercise capacity.

To localize the QTL on Chr 14, linkage analysis was performed in F _2_ mice from B6 and B6.A14 strains. Suggestive QTL were identified for both time and work at 58 cM ( Figure 3). The 1.5 LOD interval for each of these QTL spanned nearly the entire chromosome, so these QTL overlapped with previously reported QTL for pre-training and post-training work ^13^. However, the peak markers for pre-training work (4 cM) and post-training work (26 cM) QTL localize to positions distant from the QTL identified in the current study and likely represent different QTL. Further analysis using sex as an interactive covariate provided evidence for sex-specific QTL; therefore, male and female F _2_ cohorts were analyzed separately. Significant and suggestive QTL for time and work, respectively, were identified in the male cohort only and were similar to those identified in the entire cohort. Conversely, there was less evidence for exercise-related QTL on Chr 14 in female mice. This is somewhat surprising given the differences between B6 and B6.A14 female mice were comparable to those in male mice ( Figure 1). However, the peak marker for the suggestive QTL for work in the female F _2_ cohort is in close proximity to a syntenic human region linked to maximal oxygen consumption in the sedentary state in the HERITAGE Family Study ^15^. We previously reported a significant effect of sex on exercise capacity after 4 weeks of exercise training and the responses to training in mice ^13^. Sex-specific QTL also have been reported for voluntary wheel running ^28^ and exercise-related traits such as muscle mass ^29, 30^. Furthermore, Wang *et al.* identified several QTL related to fat mass in the mouse which were influenced by sex ^31^. Global gene expression analysis of liver tissue in the same population of mice revealed that a large percentage of expression QTL also were influenced by sex. These data suggest that sex can affect the genetic regulation of gene expression as well as clinical phenotypes. Therefore sex-specific affects should be considered when investigating the genetic regulation of phenotypes, especially those such as exercise capacity that are known to differ between males and females.

One potential explanation for the limited evidence for exercise QTL in the F _2_ cohort is that the number of animals was insufficient for detecting multiple QTL with small effects. However, the number of mice included in the entire F _2_ cohort or each sex-specific cohort is comparable to most intercross populations utilizing a CSS and B6 strains and should have been sufficient to detect at least 1 QTL ^16, 24, 25^. Similar to the current study, Burrage *et al.* were also unable to localize QTL in CSS × B6 intercross populations for several traits showing significant differences between parental CSS and B6 mice ^25^. They concluded that multiple QTL with opposing effects might be present on individual chromosomes and that congenic strains might be more advantageous for QTL detection and mapping than larger intercross populations. Alternatively, a close inspection of the allelic effects for each exercise QTL suggests that alleles derived from the A/J stain contribute to increasing exercise capacity ( Table 3). This was most evident in the female F _2_ cohort. The suggestive QTL for work identified in this population mapped to a position (16 cM) that was different from that observed in the entire F _2_ and male-only cohorts. In females, mice homozygous for the parental A allele performed significantly greater work that mice homozygous for the parental B allele. Heterozygous mice were intermediate and not significantly different from either parental genotyping suggesting an additive inheritance pattern with the A allele conferring increasing exercise capacity. In the full F _2_ and male-only cohorts, there was no significant difference between mice homozygous for the parental genotypes and heterozygous mice had the highest exercise capacity. Thus, at some locations A and B alleles can interact to elicit a phenotype greater that either parental genotype.

Collectively, these data support the use of CSS as a model for the genetic analysis of exercise capacity. They also provide evidence that genetic factors on Chr 14 contribute to the variation in exercise capacity. Based on the complexity of the exercise phenotype, a survey of the complete C57BL/6J-Chr ^A/J^/NaJ CSS panel will likely identify multiple chromosomes of interest and potential QTL for exercise capacity. Furthermore, the sex-dependent differences in exercise capacity and the putative sex-specific QTL imply that the genetic architecture underlying exercise capacity might be different between males and females. Thus, any such survey should be conducted in male and female mice to elucidate the potential genotype by sex interaction underlying differences in exercise capacity between males and females. Once strong candidate genes are identified, the link between exercise capacity and cardiorespiratory fitness, and the mechanistic basis for diseases associated with low cardiorespiratory fitness can be explored.

## Data availability

Figshare: Effect of chromosome 14 substitution on intrinsic exercise capacity in mice: R/qtl linkage analysis and phenotype data, http://dx.doi.org/10.6084/m9.figshare.893581 ^32^.
