# Supplementary material for: Effect of chromosome substitution on intrinsic exercise capacity in mice
Source: F1000Res. 2014 May 28;3:9. Originally published 2014 Jan 13. [Version 2] doi: 10.12688/f1000research.3-9.v2 (PMC4032107; doi:10.12688/f1000research.3-9.v2)
Supplement: Peer review history

# Peer review: Referee response for version 2

**DOI:** 10.5256/f1000research.4599.r3115

**Published:** 2014-06-23

**Authors:** Reuben Howden

**Affiliations:** 1 Department of Kinesiology, University of North Carolina at Charlotte, Charlotte, NC, USA

I appreciated and enjoyed the interesting conversation with these authors on an important and engaging topic.  I have no further comment regarding this manuscript.

I have read this submission. I believe that I have an appropriate level of expertise to confirm that it is of an acceptable scientific standard.

# Peer review: Referee response for version 1

**DOI:** 10.5256/f1000research.3335.r4461

**Published:** 2014-04-11

**Authors:** Reuben Howden

**Affiliations:** 1 Department of Kinesiology, University of North Carolina at Charlotte, Charlotte, NC, USA

The authors give the impression that a higher, genetically controlled, exercise capacity should equal lower disease risk based on previous work. However, I wonder if a genetic predisposition to a higher exercise capacity is more or less important that habitual exercise itself. It would be interesting to investigate disease risk in inbred mice using training programs of differing volume/intensity so the effect of environmental factors (e.g. habitual exercise) can be assessed in a controlled environment. Habitual exercise and disease risk reductions may be achievable without developing an exceptional exercise capacity. Prof. Edward Howley’s recent comments about recommending vigorous exercise, needed for high exercise capacity, from the end of his lecture ^1^ may be pertinent here.

Assessment of exercise capacity by treadmill running -  this may be an age old question, but it remains an important one; how do the authors know they successfully assessed exercise capacity in these strains and that differences in treadmill running time were not influenced by individual strain motivation to continue exercise? While this is not a study design flaw, it could change the question asked about factors influencing sedentary behavior and chronic disease risk in human populations.

As the authors point out, a limitation of their study is the small F _2_ cohort. While the reported cohort size may be concordant with previous work, that does not mean there were sufficient meiotic events to fine map their previously identified QTL on Chr14.  

It is difficult to determine from this article if the Chr14 QTL was reduced in the current study.  Perhaps the authors could provide more information about that.  

Did the use of CSS increase the authors confidence about specific candidate genes identified previously? ^2^ What potential candidate genes are located within the present (refined?) Chr14 QTL?

I agree that congenic strains might be useful for identifying which regions within the reported Chr14 QTL, although because of the likely complexity of factors influencing exercise capacity, demonstrating a phenotypic influence of more specific genomic regions or individual candidate genes may be challenging

I have read this submission. I believe that I have an appropriate level of expertise to confirm that it is of an acceptable scientific standard.

## References

[1] HowleyE: How much exercise is enough?. *Huffines Discussion*.2013; Reference source

[2] MassettMPFanRBerkBC: Quantitative trait loci for exercise training responses in FVB/NJ and C57BL/6J mice. *Physiol Genomics.*.2009;40(1) : 915-2210.1152/physiolgenomics.00116.200919789284PMC2807210

# Response

**Published:** 2014-04-23

**Authors:** Michael Massett

**Affiliations:** Texas A&M University, USA

*“The authors give the impression that a higher, genetically controlled, exercise capacity should equal lower disease risk based on previous work. However, I wonder if a genetic predisposition to a higher exercise capacity is more or less important that habitual exercise itself. It would be interesting to investigate disease risk in inbred mice using training programs of differing volume/intensity so the effect of environmental factors (e.g. habitual exercise) can be assessed in a controlled environment. Habitual exercise and disease risk reductions may be achievable without developing an exceptional exercise capacity. Prof. Edward Howley’s recent comments about recommending vigorous exercise, needed for high exercise capacity, from the end of his lecture may be pertinent here.”*

The Reviewer raises several good points in this comment. First, our goal in the Introduction was to remind the readers of the well-documented inverse relationship between cardiorespiratory fitness and morbidity and mortality in humans. This relationship appears to plateau at 10 METs, suggesting that having a fitness level above 10 METs does not confer significant additional protection (Kokkinos *et al., *2008). Therefore, we agree with the Reviewer that developing high levels of exercise capacity and/or fitness are not required to achieve beneficial effects.

Regarding the question of fitness versus physical activity, the Reviewer is correct in pointing out that increasing physical activity can reduce the risk of all-cause mortality and CVD and that some benefit may be gained by increasing activity without improving fitness (maximal oxygen uptake). However, Williams (2001) did point out that cardiorespiratory fitness and physical activity should be considered independently as predictors of disease risk and that the relative risk of heart disease in the most fit group is nearly half that of the most active group. These data imply that both fitness and activity are important factors in reducing the risk of cardiovascular disease. Determining the genetic basis for each phenotype could yield important information regarding the beneficial effects of exercise.

*“Assessment of exercise capacity by treadmill running - this may be an age old question, but it remains an important one; how do the authors know they successfully assessed exercise capacity in these strains and that differences in treadmill running time were not influenced by individual strain motivation to continue exercise? While this is not a study design flaw, it could change the question asked about factors influencing sedentary behavior and chronic disease risk in human populations.”*

In the current study, exercise performance was assessed during a graded exercise test, which was stopped when mice exhibited pre-defined behaviors that we, and others, have defined as exhaustion. As pointed out by Booth, Laye and Spangenburg (2010), in ideal settings, exhaustion would be defined by a number of physiological markers. The large number of mice required for genetic experiments exceeded our capacity to assess maximal oxygen consumption and/or blood lactate levels at the time of testing. However, Desai *et al.* (1997) showed that an exercise test similar to the one utilized in the current study elicited heart rates near 750 beats per minute and a respiratory exchange ratio at or above 1.0 in mice from several inbred strains. We cannot assess an animal’s motivation to run on the treadmill so we do not know how much this might have contributed to our results. But, as demonstrated by Lerman *et al. *(2002) the willingness to run voluntarily is likely determined by different genetic factors than those underlying the variation in intrinsic exercise capacity in the untrained state. This latter phenotype is the focus of the current study. 

*“As the authors point out, a limitation of their study is the small F cohort. While the reported cohort size may be concordant with previous work, that does not mean there were sufficient meiotic events to fine map their previously identified QTL on Chr14.”*

The number of mice utilized in this study was sufficient to detect at least one QTL explaining 5% of the variance in exercise capacity with a power of 50% (Belknap, 2003).Increasing the number of mice would likely have allowed us to detect more QTL with smaller effects. However, as the reviewer points out, an experimental model with a greater number of recombination events, such as an advanced intercross line based on A and B6 strains, the hybrid mouse diversity panel, or mice from the collaborative cross, would probably have yielded a greater resolution for our QTL interval.

*“It is difficult to determine from this article if the Chr14 QTL was reduced in the current study. Perhaps the authors could provide more information about that.”*

The QTL for pre-training work on Chromosome 14 identified in our previous study was located at 4 cM with a 95% CI of 0-38 cM. The suggestive QTL for time and work identified in the current study were located near 58 cM and the 95% CI were very large. Although the QTL in the current study did overlap with our previously identified QTL, it is very likely that the QTL identified in the current study are different from our previous report. A brief statement regarding this issue is located in the fourth paragraph of the Discussion section.

*“Did the use of CSS increase the authors confidence about specific candidate genes identified previously? What potential candidate genes are located within the present (refined?) Chr14 QTL?”*

Because there was no overlap between the current and previous QTL, the use of the CSS model did not increase our confidence regarding specific candidate genes in the Chromosome 14 QTL region. However, given the complexity of the exercise capacity phenotype, performing a genome scan using a complete set of CSS might be a logical step toward identifying chromosomes of interest and potential candidate genes. These chromosomes could then be targeted for more detailed analyses.

*“I agree that congenic strains might be useful for identifying which regions within the reported Chr14 QTL, although because of the likely complexity of factors influencing exercise capacity, demonstrating a phenotypic influence of more specific genomic regions or individual candidate genes may be challenging”*

We agree with the Reviewer that identifying candidate genes for exercise and exercise-related traits is challenging. However, the availability of resources for genetic and genomic studies continues to increase, which should facilitate discovery of genes and gene networks that regulate variation in exercise capacity, fitness, and potentially diseases associated with low levels of fitness.

# Peer review: Referee response for version 1

**DOI:** 10.5256/f1000research.3335.r3576

**Published:** 2014-03-31

**Authors:** Michael Turner

**Affiliations:** 1 Department of Kinesiology, University of North Carolina at Charlotte, Charlotte, NC, USA

This manuscript is very well written regarding a strong, well thought out research design.  The research is cutting edge and moves the knowledge regarding possible genetic factors influencing intrinsic exercise capacity in active mice.  Chromosome 14 is an important participant in this phenotype and the authors do an excellent job utilizing chromosome substitution to continue pursuing their research interests.  

Minor comments: 

- The authors point out in the first paragraph of the Results that body mass was less in the B6.A14 mice compared to the B6 mice. Could the authors perform a simple regression to assess the influence of differing body mass on intrinsic exercise capacity as a potential confounding factor to their findings?  
- The third sentence in the second paragraph of the Results appears to conflict with the previous statement regarding differences in work with sex in the F _2_ generation. If I'm reading these two sentences incorrectly I would ask a clearer statement be made to help the reader understand the sex-related difference in work with these F _2_ mice.
- In the last few sentences in the fourth paragraph of the Discussion the authors present an interesting discussion regarding the different QTLs on chromosome 14 that may be playing a role in the male vs. female F _2_ mice and intrinsic exercise capacity.  More discussion would add to the major findings and future directions for readers.  This is a difficult research issue and a bit more discussion would direct the readers towards an appreciation of this position.  Possibly a better transition to the argument provided in the following paragraph would assist in the interpretation of the authors' findings.

I have read this submission. I believe that I have an appropriate level of expertise to confirm that it is of an acceptable scientific standard.

# Response

**Published:** 2014-04-23

**Authors:** Michael Massett

**Affiliations:** Texas A&M University, USA

*The authors point out in the first paragraph of the Results that body mass was less in the B6.A14 mice compared to the B6 mice. Could the authors perform a simple regression to assess the influence of differing body mass on intrinsic exercise capacity as a potential confounding factor to their findings?*

We thank the Reviewer for this suggestion. We performed a linear regression to determine the relation between body mass and exercise capacity expressed as time.  We analyzed each of the inbred strains separately as well as the F _2_ population. For each of these groups, exercise time was significantly negatively correlated with body mass. In general, body mass explained 50% to 74% of the variance in exercise time in the inbred strains. In the F _2_ population, the variance in exercise time explained by body mass was approximately 54%; however, this percentage decreased when the population was divided by sex. In males this percentage was 22 % and in females, less than 2%. We will include the correlation data in the revised manuscript to identify both the direction and magnitude of this relation.

*The third sentence in the second paragraph of the Results appears to conflict with the previous statement regarding differences in work with sex in the F generation. If I'm reading these two sentences incorrectly I would ask a clearer statement be made to help the reader understand the sex-related difference in work with these F _2_ mice.*

We thank the Reviewer for pointing out this confusing description. Simply stated, work is determined as the product of exercise capacity and body mass. Female mice ran longer/farther than male mice, but weighed less; therefore work was approximately equal between male and female F _2_ mice. We will edit this description in the revised manuscript.

*In the last few sentences in the fourth paragraph of the Discussion the authors present an interesting discussion regarding the different QTLs on chromosome 14 that may be playing a role in the male vs. female F mice and intrinsic exercise capacity. More discussion would add to the major findings and future directions for readers. This is a difficult research issue and a bit more discussion would direct the readers towards an appreciation of this position. Possibly a better transition to the argument provided in the following paragraph would assist in the interpretation of the authors' findings.*

We are grateful for the Reviewer’s appreciation of the complexity of the phenotype and the number of genetic and environmental factors that influence this phenotype. We will expand our discussion of some of these genetic factors in the revised manuscript.
